# Blunt chest trauma caused rupture of the left main coronary artery in a 15 year old motocross rider

**DOI:** 10.1186/1749-8090-8-S1-P25

**Published:** 2013-09-11

**Authors:** I Burcar, M Petricevic, H Gasparovic, V Coric, B Biocina

**Affiliations:** 1 Department of Cardiac Surgery, University Hospital Rebro, Zagreb, Croatia; 2 Division of Cardiac Surgery, University Hospital Center Split, Split, Croatia

## Case report

A 15 year old man suffered chest trauma as a participant of the international motocross race. After falling of the motorcycle, he was run over his chest by another diver. He was unconscious, Glasgow Coma Score was 5 and eye pupils did not react. Resuscitation with sedation, relaxation, analgesia and endotracheal intubation with mechanical ventilation were performed in the emergency vehicle. Mild hypotension and microhemathuria was present. Blood coming from the airways was aspirated periodically. Physical examination revealed excoriations and haemathomas of the face, lower left hemithorax and extremities were noticed. CT scan of the head, thorax and abdomen was performed- bilateral contusion of the lung was revealed. On the ECG ST-segment denivelation is noticed. Troponin, lactate dehydrogenasis (LDH) and creatin phosphate kinaze (CPK) levels were elevated. Transthoracic echocardiogram (TTE) revealed 10 mm thick pericardial effusion. There are no pathologic findings on ECG. That was indication for emergent cardiac surgery operation. After conventional median sternotomy and pericardiotomy was performed, 150 ml of blood was evacuated from pericardial space. Patient was placed on cardiopulmonary bypass (CPB) with bicaval venous and aortic cannulation. After crossclamping aorta MPA was transected to approach LMCA. Damage of intimal layer of the MPA was found, originating just above the pulmonary valve and ending at bifurcation of pulmonary artery. After aortotomy the probe was inserted in the lumen of the LMCA. Subepicardial haemathoma of the left ventricle was found just next to LMCA. Defect of the LMCA wall was not suitable for reconstruction because its size was too big. So ligation of the left anterior descending artery (LAD) and circumflex artery (Cx) and coronary artery bypass (CABG) was performed. LAD was bypassed with left internal thoracic artery (LITA) and Cx with saphenous conduit. After aortotomy was sutured and clamp removed, MPA was closed. On transesophageal echosonography, ishemia of the anterior wall of the LV was noticed. We tried to wean the patient from CPB but it was unsuccessful. Patient died because of the left heart failure following irreversible myocardial damage following ischemia.

